# Cost-of-illness of type 2 diabetes mellitus in low and lower-middle income countries: a systematic review

**DOI:** 10.1186/s12913-018-3772-8

**Published:** 2018-12-17

**Authors:** Afsana Afroz, Mohammed J. Alramadan, Md Nassif Hossain, Lorena Romero, Khurshid Alam, Dianna J. Magliano, Baki Billah

**Affiliations:** 1 Department of Epidemiology and Preventive Medicine, School of Public Health and Preventive Medicine, Monash University, Melbourne, Australia; 2 The Ian Potter Library, The Alfred, Melbourne, Australia; 3 Murdoch Childrens Research Institute, Melbourne, Australia; 4 BakerIDI Heart and Diabetes Institute, Melbourne, Australia

**Keywords:** Cost-of-illness, Direct cost, Indirect cost, LMICs, Systematic review, Type 2 diabetes mellitus

## Abstract

**Background:**

Diabetes is one of the world’s most prevalent and serious non-communicable diseases (NCDs). It is a leading cause of death, disability and financial loss; moreover, it is identified as a major threat to global development. The chronic nature of diabetes and its related complications make it a costly disease. Estimating the total cost of an illness is a useful aid to national and international health policy decision making. The aim of this systematic review is to summarise the impact of the cost-of-illness of type 2 diabetes mellitus in low and lower-middle income countries, and to identify methodological gaps in measuring the cost-of-illness of type 2 diabetes mellitus.

**Methods:**

This systematic review considers studies that reported the cost-of-illness of type 2 diabetes in subjects aged 18 years and above in low and lower-middle income countries. The search engines MEDLINE, EMBASE, CINAHL, PSYCINFO and COCHRANE were used form date of their inception to September 2018. Two authors independently identified the eligible studies. Costs reported in the included studies were converted to US dollars in relation to the dates mentioned in the studies.

**Results:**

The systematic search identified eight eligible studies conducted in low and lower-middle income countries. There was a considerable variation in the costs and method used in these studies. The annual average cost (both direct and indirect) per person for treating type 2 diabetes mellitus ranged from USD29.91 to USD237.38, direct costs ranged from USD106.53 to USD293.79, and indirect costs ranged from USD1.92 to USD73.4 per person per year. Hospitalization cost was the major contributor of direct costs followed by drug costs.

**Conclusion:**

Type 2 diabetes mellitus imposes a considerable economic burden which most directly affects the patients in low and lower-middle income countries. There is enormous scope for adding research-based evidence that is methodologically sound to gain a more accurate estimation of cost and to facilitate comparison between studies.

**Electronic supplementary material:**

The online version of this article (10.1186/s12913-018-3772-8) contains supplementary material, which is available to authorized users.

## Background

Globally, diabetes is one of the most prevalent growing epidemics that causes premature death, disability, and economic loss; moreover, it is a significant threat to global development [1–4]. In 2015, 415 million people were diagnosed with diabetes mellitus (DM) and the number is predicted to be 624 million by 2040 [5]. This global increase in DM is attributed to population growth, ageing and an increasing trend towards unhealthy diets, sedentary lifestyles and obesity [6]. In 2013, about two-thirds of all individuals with diabetes lived in the lower-middle-income countries (LMICs) [4]. Its increased prevalence in the LMICs appears to be fuelled by rapid urbanisation, change in diet and increasingly sedentary lifestyles [7]. Diabetes is linked to macrovascular complications such as: cardiovascular, cerebrovascular, and peripheral vascular diseases, as well as microvascular complications such as: retinopathy, nephropathy and neuropathy [8, 9]. DM is a costly diseases due to its prolonged nature and associated complications [10]. Global healthcare expenditure to treat and prevent diabetes and its associated complications was USD376 billion in 2010; the expenditure is projected to be USD490 billion in 2030 [11].

Cost-of-illness (COI) studies include of the impact of a disease on individuals, community and the country as a whole from various aspects. The aim of a COI study is to identify and quantify all costs of a particular disease including direct, indirect and intangible costs. The output is the estimation of the total economic burden of a specific diseases to the society in monetary terms [12]. It is commonly accepted that estimating the total cost of an illness is a useful tool to national and international health policy decision making [13].

Development or improvement of cost-effective solutions for the management of type 2 diabetes mellitus (T2DM) depends on costs identification. A few studies addressed the COI of T2DM in LMICs, however no systematic review has been conducted. A systematic review helps to summarise the findings, discuss the methods used and identify the gaps in previous studies. Thus, the aim of this study was to perform a systematic review on COI of T2DM in LMICs which will summarise the previous findings and provide recommendations for appropriately measuring the economic burden of T2DM in future studies.

### Overview of cost-of- illness studies

The COI typically stratifies costs into three categories: direct, indirect and intangible costs.

#### Direct cost

The direct costs associated with resources used to treat the disease including expenditure for medical care and treatment. The direct cost covers two sub categories: (a) direct medical costs which include hospitalisation costs, outpatient visits, medications, laboratory tests, medical supplies, emergency services, and traditional medicine services; and (b) direct non-medical costs includes travel and meal cost en-route to the hospital.

#### Indirect cost

The indirect cost includes the opportunity cost of productive time loss by patients and their accompanying persons due to morbidity, disability or premature death.

#### Intangible cost

The intangible cost includes social, emotional and human costs. Since the costs cannot be converted to money, they are unmeasurable. Intangible cost includes pain, suffering, loss of quality of life, lack of participation in social events or poor emotional health.

### Perspective of COI studies

There are different perspectives to consider when undertaking a COI study, and each category includes marginally different sets of cost items. Thus, each perspective finally leads to different kinds of results for the same diseases. These perspectives can be societal, the health care system, third-party payers, business, the government, and participants and their families [12, 14, 15]. The societal perspective, which covers the maximum components of cost, is generally the most preferred. Table 1 presents the cost categories included in each perspective.

**Table 1:** Costs included in cost-of-illness studies by perspective

Perspective	Medical cost	Morbidity cost	Mortality cost	Transportation/Nonmedical cost	Transfer payment
Societal	All costs	All costs	All costs	All costs	–
Health care system	All costs	–	–	–	–
Third-party payer	Covered costs	–	Covered costs	–	–
Business	Covered costs (self-insured)	Productivity loses (absenteeism)	Productivity loses	–	–
Government	Covered (medical aid)	–	–	Criminal justice costs	Attributable to illness
Participants and families	Out-of-pocket costs	Wage losses/ Household production	Wage losses/ Household production	Out-of-pocket costs	Amount received

Source: Luce et al. 1996. 37 [15]

### Approaches of COI studies

The approach of a COI study can be either prospective or retrospective. In a retrospective approach, all relevant cost components are followed up from previous records; thus, it is less expensive and less time consuming. On the other hand, in a prospective approach, all relevant cost components are followed up for the coming year means collecting data from patients over time; thus, it is quite expensive in terms of time and resources.

### Types: Incidence-based vs. prevalence-based

There are two different types of COI studies—prevalence-based and incidence-based, depending on in which way the data are being used. The prevalence method estimates the economic burden of a diseases for a specific period, commonly six months to a year. Conversely, the incidence-based approach estimates the life-time cost, from the onset of diseases to cure or death. The prevalence-based method is the most common. Both the prevalence-based and incidence-based COI studies can be designed either in a prospective or retrospective way [16].

## Methods

### Criteria for selection of the studies

A systematic search was conducted in the MEDLINE, EMBASE, CINAHL, PHYCINFO and COCHRANE databases. The search (Additional file 1) has covered the period from date of their inception to September 2018. The search was limited to studies that addressed COI of T2DM in LMICs and published in English language. Furthermore, a manual search was conducted to identify relevant articles in the reference lists of the identified articles.

In the present review, a systematic search was conducted to screen for relevant articles following the pre-set inclusion and exclusion criteria. Results of original studies conducted in LMICs in English covering COI, healthcare cost, or resource use for T2DM were included in this review. The following studies were excluded from this review: studies conducted on other types of the diabetic population, studies that included a cost-effectiveness analysis of intervention, drug or treatments, studies performed on animals or in vitro studies, and studies conducted in high income countries. Also, review papers, conference abstracts, case reports, editorials and letters to the editor were excluded.

### Screening and data extraction

A comprehensive search strategy developed with the help of an expert research librarian was implemented combining the three concepts: cost, T2DM and LMICs. A two-tire screening process was followed to retrieve the relevant articles from the literature search. Two trained, independent reviewers helped in the process of selecting relevant articles. Firstly, the titles of articles were screened under the following terms: for “**cost**” or similar words such as “cost or expenditure” or “costs and cost analysis” or “health care costs” or “cost of illness”, and for “**diabetes”** similar words such as “type 2 diabetes,” or “niddm or t2dm,” or “non-insulin dependent diabetes mellitus” were included as a search term. The final search was conducted combining the three concepts using “AND”. Secondly, two reviewers independently evaluated the abstracts and, if necessary, they read the full texts of all the articles which were selected considering the selection criteria. Any disagreement between the two reviewers was finalised upon discussion and additional consultancy with a third reviewer, if necessary. The reference lists of all the selected articles were screened manually for additional citations. Important information on the study methods and key findings were then retrieved from the articles and summarised in Tables 2 and 3. To compare different currencies that have been used in the studies, all costs were converted to PPP (Purchasing Power Parity). Local currencies were inflated applying the World Bank’s consumer price index (CPI) [17] to make them equivalent to the cost in 2012, and then converted into US dollars (USD) using a conversion software [18].

**Table 2:** Characteristics of cost-of-illness studies for type 2 diabetes mellitus, arranged by year and reflecting costs incurred in the year stated

Sl no	Author (Year of costing)	Data source	Sample size	Study design	Country	Direct	%	PPP 2012	Indirect	%	PPP 2012
1	Shobana et al. [24] (1997)	Hospital (PHD & GGD)	596	Retrospective	India	Median annual direct cost for PHD Rs.4510 GGD Rs.247		PHD 218.7 GGD 11.9			
2	Ramachandran et al. [23] (2005)	Hospitals, clinics, (Rural & Urban)	556	Retrospective	India	Median annual direct cost for urban Rs 10,000, rural Rs 6260		Urban 323.5 rural 202.5			
3	Kumar et al. [25] (2005^)^	Community	819	Retrospective	India	Mean direct cost Rs. 6000		194.1			
4	Khowaja et al. [29] (2005)	Clinics (P,G&NGO)	345	Retrospective	Pakistan	Mean direct cost Rs 11,580		270.9	Meanproductivity loss by participants Rs. 113 and attendants was Rs. 208		2.6 4.8
5	Tharkar et al. [26] (2009)	Community	718	Retrospective	India	Median annual direct cost Rs. 25,391	83.70	605.3	The median annual indirect cost 4970 INR	16.3	118.5
6	Chandra et al. [27] (2012)	Hospital	219	Retrospective	India	Average annual direct cost Rs 8822	69.08	163.9	Average annual indirect cost Rs. 3949	30.9	73.4
7	Suleiman et al. [30] (2011–2012^)^	Hospital	321	Retrospective	Nigeria	Average annual direct cost NG 45531.19		284.5			
8	Akari et al. [28] (2012)	Hospital	150	Prospective observational	India	Average annual direct cost with complication US$293.79 without complication US$27.7	98.8 92.6	293.7 27.7	Average annual indirect cost with complication US$20.34 without complication US$2.21	1.1 7.4	20.3 2.2

Note: PPP = Purchasing Power Parity. Local currencies were inflated applying the World Bank’s consumer price index (CPI) to make them equivalent to the cost in 2012, and then converted into USD as PPP applying the conversion rate on 3oth June 2012 using 1USD = 55.9 Indian Rupee, 94.9 Pakistan Rupee, and 160.0 Nigerian naira

**Table 3:** Components of direct healthcare costs for diabetes mellitus

Sl	Author	Year of Publication	Country	Hospital inpatient	Physician services	Emergency outpatient	Drugs	Laboratory tests	Other health professional/allied health	Daily self-management	Transport	Food on the way to hospital	Dietary management	Surgery	Subsidized consultation and investigation cost	Informal care/caregiver	Indirect cost	Intangible cost	National estimation
1	Shobana et al. [24]	1999	India	√	√		√	√			√			√					√
2	Ramachandran et al. [23]	2007	India	√	√		√	√						√					
3	Kumar et al. [25]	2008	India		√		√	√		√									
4	Khowaja et al. [29]	2007	Pakistan		√		√	√			√	√			√		√		
5	Tharkar et al. [26]	2010	India	√	√		√	√		√	√	√				√	√	√	√
6	Chandra et al. [27]	2014	India		√		√	√						√			√		
7	Suleiman et al. [30]	2014	Nigeria		√		√	√			√								√
8	Akari et al. [28]	2013	India	√	√		√	√			√	√					√		

### Critical review of data and quality of studies

The quality of the studies was critically assessed following the previous reviews and guidelines [19–22]. The assessment tool (Additional file 1) had 15 indicators and each indicator was rated as high with a score of 1, partial with a score of 0.5 and low with a score of 0. The maximum obtainable score was 17. The quality assessment was also done by two independent authors; there was no significant disagreement between the authors.

## Results

**Fig. 1:**
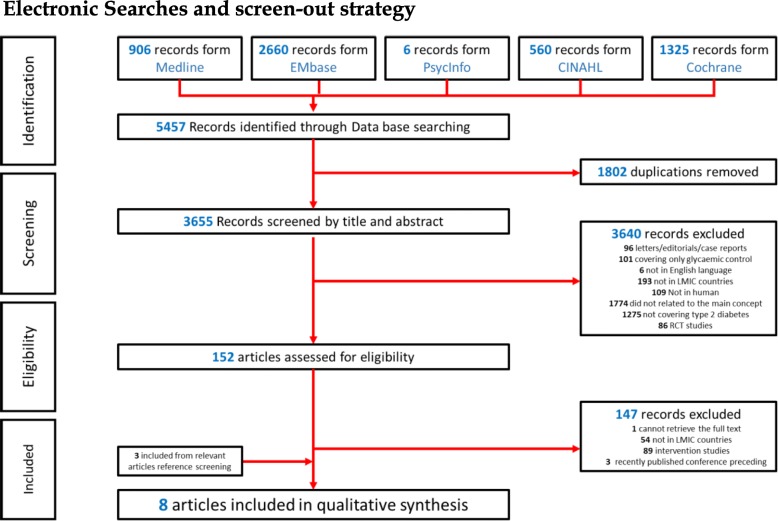
PRISMA flow chart through the different phases of the systematic review

The initial search resulted in 5457 articles. Of these, 1802 were duplicates and were removed. Of the remaining 3655 articles, 3640 were excluded by screening of abstracts and titles, leaving 152 articles for full-text review. After screening the full-text articles, 147 were excluded leaving only five articles. Then the reference lists of these five articles were screened manually, and an additional three articles were included. Subsequently, eight articles were finally selected for inclusion in this systematic review (Fig. 1).

### Characteristics of the studies

Table 2 summarises the methods and key findings of the eight articles included in the systematic review. Two-thirds (*n* = 6) of the studies were conducted in India [23–28], one (*n* = 1) in Pakistan [29] and one (n = 1) in Nigeria [30]. Four of the eight studies estimated both direct and indirect costs of T2DM [26–29] while the other half estimated only direct costs. Only one [26] study reported intangible costs. Furthermore, only three studies generalised the estimated cost for the national population [24–26]. All the studies were cross-sectional retrospective, except the study by Sadanandam et.al which was a prospective observational study. Only two [25, 26] of the eight studies recruited the study participants from the community while in the remaining studies data were collected from different levels of the hospitals.

### Cost components

There was a variation among the cost components that estimated the direct and indirect cost of the studies (Table 3). All the studies considered costs associated with hospitalisation, physician services, laboratory tests and drugs as components of direct cost. All reported direct costs were calculated from a patient’s perspective and all indirect costs were measured using a human capital approach [31].

### Total cost of T2DM

Of the eight articles in this review, 50% (*n* = 4) of the studies [26, 28–30] estimated the total cost (both direct and indirect costs). The annual average cost (both direct and indirect) per person for treating T2DM ranged from USD29.91 to USD237.38. However, Tharkar et al. [26] reported the annual average cost as USD672.6 per person, which included costs associated with complications.

### Direct, indirect and intangible costs of T2DM

**Fig. 2:**
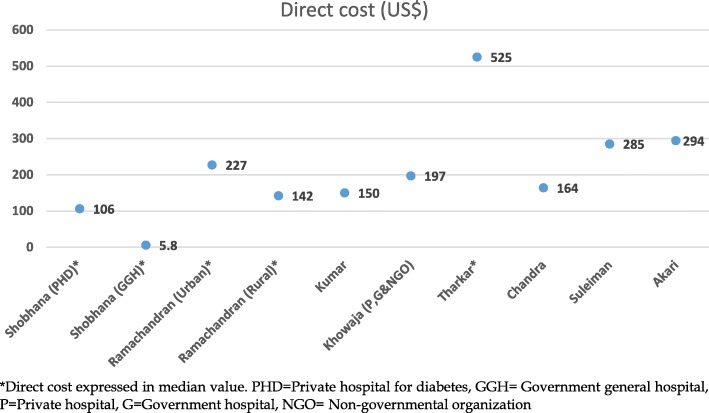
Total direct cost of T2DM by reported studies

The reported costs components of T2DM varied by countries and year (Table 1). Most studies reported the average (mean) annual cost while three reported the median cost [23, 24, 26]. A clear trend of increment of the average annual direct and indirect costs per person per year (PPPY), respectively, from USD106.53 in PPP (Purchasing Power Parity) 2012 to USD293.79 and from USD2.6 in PPP 2012) to USD73.4 was observed between 1997 and 2012. Only one study reported intangible cost, which was USD41.1 PPPY. Furthermore, direct costs were higher compared to indirect costs for the four studies that estimate both direct and indirect costs. Hospitalisation costs were predominant among the contributors of direct costs, which were about half of the total costs. In the studies that did not consider hospitalisation costs, medication costs were the highest contributor of direct costs, which accounted for 26–66% of the total cost [26, 28]. The total direct costs ranged from approximately USD194.1 in PPP 2012 to USD314 (Fig. 2). Shobana et al. [24] reported that the minimum direct cost was USD5.8 in a government general hospital (GGH) setting. The minimum direct cost USD11.9 in PPP 2012 was low because 70% of the GGH subjects were unemployed and were from lower socio-economic groups. In contrast, in a private hospital setting the total direct cost was USD218.7 in PPP 2012 as most subjects were from middle, upper-middle and higher socio-economic groups. Tharkar et al. reported [26] a median direct cost of USD605.3 in PPP2012, which may be reflected by the fact that this study considered the highest number of cost components among the studies in this review.

### National burden of T2DM

The study by Shobhana et al. [24] in 1997 estimated an annual direct cost of USD2.2 billion (USD4.3 billion, in PPP 2012) for diabetes health care for approximately 20 million diabetic patients in India. Tharkar et al. [26] projected the total annual cost (direct and indirect costs) of USD31.9 billion (USD36.7 billion in PPP 2012) for type 2 diabetes in India in 2010. Ismail et al. [30] reported that in Nigeria, the annual average direct cost was USD284.56 PPPY for T2DM in 2012; this study also showed that without any complication the cost was USD1.64 billion.

### Associated factors for costs

Of the eight studies in this review, two [27, 29] described the factors associated with the costs using bivariate analysis and three [23, 25, 26] described the factors associated with the direct cost using multivariable analysis. Results of the bivariate analysis showed that age, number of complications and duration of diabetes were associated with direct costs. In the multivariable analysis, education, income, number of complications, duration of diabetes, mode of treatment, hospitalisations and surgery were found to be associated with direct costs after adjustment for other potential variables.

### Quality analysis

To execute a good COI analysis, it is necessary to perform quality assessment focusing on the important components. The studies included in this review were evaluated on a 17-point scale. The median score was 8.5 (range: 5 to 11.5). The studies in the review lacked precise definition and type of diabetes. Only three articles clearly defined diabetes and its type. No studies stated a clear definition of co-morbidity or complications they considered. Most studies were conducted over a satisfactory period of 6 months, however, two studies did not mention any timeline [27, 30].

Fifty percent of the studies included a reasonable number (*n* = 500 to 800) of participants while the remaining studies were based on samples which were either too small or limited to a specific group of people. Additionally, it is notable that the included studies commonly focused on the middle-income and high-income population. All the studies collected data from individuals based on self-assessment of costs using a questionnaire. Interestingly, half of the studies verified the self-assessment information by the actual bill or records collected from the care providers.

The quality assessment also considered the suitability of the different components of costs that were used. The suitability of cost components in each included study was evaluated against the study objectives and the minimum requirements of a comprehensive COI study: cost components, perspective, approach, data source and use of sensitivity analysis [22, 32]. Only four studies included the appropriate costs.

Considering the methods, most studies did not include enough details of the methods they used. Particularly, 50% of the studies did not refer to how they calculated the costs. None of the studies applied the incremental costs method and only two studies used the regression methods. The review of results indicates that the prevalence-based bottom-up approach was common between the studies. Only two studies performed the uncertainty test via sensitivity analysis and three studies conducted linear or multivariable regression analysis.

Besides inconsistencies in the type and amount of information provided regarding methods, limitations were not discussed in some studies. Five studies comprehensively discussed limitation on data, components of cost, study assumptions and methods.

In terms of the statistical analysis that has been used, three studies performed all necessary statistical analyses. The remaining five studies employed the Student t-test, χ^2^-tset, or the Kruskal Wallies or Mann Whitney U-test to determine the statistical significance. One study did not perform any statistical analysis. Seven studies presented the average cost either by mean along with standard deviation or median with range while one study included only the mean.

## Discussion

Type 2 DM imposes a large economic burden on the health care system, society, and individuals. This is the first systematic review, which appraises the COI of T2DM in LMICs only. The most notable finding of this review is that a wide variation in methodology was used to estimate the COI and the cost components. This review shows that most studies have focused on a subset of the population or health-care expenditures. The quality of the studies reviewed were deemed to be fair.

The choice of cost-estimation methodology was largely driven by the availability of data which greatly influenced the magnitude of cost estimates. All the studies in this review were conducted on a relatively small sample and most of them were based on a single-centre. In the LMICs, self-administered surveys employing patients’ perspective was the most popular method to accumulate data on the cost of diabetes and the sample-size, ranging from 150 [28] to 819 [25]. Hence, they were mostly limited to a specific group of people (a hospital or state), and were generally representative of that particular diabetes population only rather than at the national level. National or regional level databases are scarce in LMICs and collection of relevant data requires resources that are limited in these countries too. Conversely, in the high income countries (HICs), health-care system perspective was commonly used where insurance databases and healthcare providers are the main source of cost-related information [33–40]. Thus, the sum-all medical cost approach is the commonest in HICs as all the components of costs are available in the database for people with diabetes. In HICs, the sample-size mostly varies from 1000 to several millions. As a result, these studies are likely to be representative, either at a national or regional level.

In LMICs, individual and families with diabetes often suffer with the financial burden of the illness; consequently, they are in need of better health care coverage to deal with the issue. All the studies showed that the expenditure incurred for the direct costs was met by patients in LMICs, as health insurance is not yet common and there is a lack of publicly-available medical services. Conversely, in developed countries, health care facilities for diabetes are highly structured and almost fully funded by the state.

The magnitude of cost estimates was influenced by the cost components considered in a study. The costs of consultation, laboratory test and drugs were common between the studies. The magnitude of the cost increased as the number of cost components increased in a study. Studies that were limited to fewer categories of cost components are likely to have underestimated the actual costs of T2DM. In the studies of this review, the healthcare cost components including inpatient, outpatient and medication were similar to the cost reported by earlier reviews [10, 41]. Despite the fact that indirect costs far exceed direct costs [41], this review showed that the direct cost was higher than the indirect cost. This may be attributed due to accounting for hospitalisation cost which is consistent with other global COI studies [39, 42–45]. Studies which considered cost of hospitalisation showed that it consumed almost half [26, 28] of the total cost; these findings are similar to a study conducted in the U.S.A. [46].

Three of the eight articles in this review addressed the cost of comorbidities or complications and have shown that the cost was significantly higher for patients with comorbidity or complications [26, 28, 29]. Similarly, Ng et al. suggested that a considerable amount of DM accredited healthcare expenditure is related to complications. Further, it was reported that there was more than a two-fold increase in cost for patients with complications compared to patients without complications [41]. Thus, it is important to control the catastrophic spending due to complications to reduce the burden of high out-of-pocket expenditure.

Most of the studies in this review failed to achieve the aim of a COI study due to a poor study methodology. First of all, the studies had a lack of referring to a comprehensive list of unit costs. Secondly, the cost components and the sources of data were not clearly justified, and were not even discussed as limitations of the study which raises concern about the quality of the studies. The absence of these features has made these studies less accurate in terms of data collection and costs calculation [32].

The use of disease-attributable approaches via case matching or regression analysis would be more accurate to obtain the exact estimates of the costs as suggested by Ettaro et al. [10]. This type of analysis gives more precise estimates of the economic burden of type 2 diabetes in the absence of randomised clinical trials. None of the studies incorporated disease-attributable approaches and only three studies [23, 25, 26] used regression analysis to identify the factors associated with cost.

The strength of the present study is that it incorporated a systematic search and used the recommended methodology for a review study. To avoid the potential bias in the selection of the study a dual search by two independent reviewers was done following a comprehensive inclusion and exclusion criteria. The present review provides the economic burden of T2DM in LMICs. As the focus and the methodologies of the studies reviewed were heterogeneous, we were unable to perform a meta-analysis. In addition, since the present review was limited to T2DM-related cost only, some information may differ from the studies that included both type 1 and type 2 diabetes.

## Conclusion

It is anticipated that T2DM poses a significant financial burden on the healthcare system as well as on the individual and society as a whole. In LMICs, large scale national or sub-national level studies that involve methodologically sound economic analysis are required. Evidence of the studies will be helpful for public health policymakers to establish health care priorities and allocate scarce resources to assist people with type 2 diabetes. A COI study should consider a discussion referencing the issues that might affect the cost estimates.

The explanation of all data sources will be beneficial for the researcher to replicate and improve future COI studies. Partial inclusion of all affected cost components and a relatively small sample underestimate the COI and thus limit the generalisability of the findings. Disclosure of the year that costs were valued would help to interpret findings as it is important to compare findings between different studies or populations.

At the very least, researchers should disclose pertinent details (e.g., age group, type of diabetes, area of residence) in the method and follow the consensus opinion of the experts about what should ideally be included in a COI study [32, 47]. Standardised COI will permit public health policymakers and the general population to understand the magnitude of the financial burden, to derive decisions about future benefits, to control the disease, and develop programs to improve the health of people with type 2 diabetes.

## Additional files

Additional file 1: **Appendix 1.** Search strategy. **Appendix 2.** Quality assessment tool. (DOCX 27 kb)

## Abbreviations

COI: Cost-of-illness
DM: Diabetes mellitus
LMICs: Lower-middle-income countries
NCDs: Non-communicable diseases
T2DM: Type 2 diabetes mellitus
