# Supplementary material for: Cost-of-illness of type 2 diabetes mellitus in low and lower-middle income countries: a systematic review
Source: BMC Health Serv Res. 2018 Dec 17;18:972. doi: 10.1186/s12913-018-3772-8 (PMC6296053; doi:10.1186/s12913-018-3772-8)
Supplement: Supplementary file 1 — Appendix 1. Search strategy. Appendix 2. Quality assessment tool. (DOCX 27 kb) [file 12913_2018_3772_MOESM1_ESM.docx]

**Supplementary material**

**Appendix 1** Search strategy

**MEDLINE**

| [# ▲](http://ovidsp.tx.ovid.com.ezproxy.lib.monash.edu.au/sp-3.31.1b/ovidweb.cgi?&S=KLIPFPKNAHDDLEJGNCEKDBLBPMJNAA00&Sort+Sets=descending) | **Searches** |
| --- | --- |
| 1 | (cost* or economic*).mp. |
| 2 | Diabetes Mellitus, Type 2/ or Hyperglycemia/ |
| 3 | hyperglyc*.mp. |
| 4 | (niddm or t2dm).mp. |
| 5 | (insulin resistance or insulin resistant).mp. |
| 6 | T2D.mp. |
| 7 | ((adult-onset or ketosis-resistant or maturity-onset or non-insulin-dependent or noninsulin-dependent or slow-onset or type 2 or type ii) adj1 diabet*).mp. |
| 8 | (glucose or blood sugar or glyco* or glyca* or glyce*).mp. |
| 9 | ("hb a1*" or "hba1*" or hemoglobina1* or hemoglobin a1* or haemoglobina1* or haemoglobin a1*).mp. |
| 10 | 2 or 3 or 4 or 5 or 6 or 7 |
| 11 | 8 or 9 |
| 12 | 1 and 10 and 11 |
| 13 | Developing Countries/ |
| 14 | (Afghanistan* or Albania* or Algeria* or Angola* or Argentina* or Armenia* or Azerbaijan* or Bangladesh* or Belarus* or Beliz* or Benin* or Bhutan* or Bolivia* or Bosnia* or Herzegovin* or Botswan* or Brazil* or Bulgaria* or Burkina* or Burundi* or Cabo Verde* or Cape Verde* or Cambodia* or Cameroon* or Central African or Chad* or China or Chinese or Colombia* or Comor* or Congo* or Costa Rica* or Cote d'Ivoir* or Ivory Coast or Cuba* or Djibouti* or Dominica* or Ecuador* or Egypt* or El Salvador* or Eritrea* or Ethiopia* or Fiji* or Gabon* or Gambia* or Georgia* or Ghana* or Grenad* or Guatemala* or Guinea* or Guyan* or Haiti* or Hondura* or Hungar* or India* or Indonesia* or Iran* or Iraq* or Jamaica* or Jordan* or Kazakhstan* or Kenya* or Kiribati* or Korea* or Kosov* or Kyrgyz Republic or Lao* or Leban* or Lesotho* or Liberia* or Libya* or Macedonia* or Madagascar* or Malawi* or Malaysia* or Maldiv* or Mali* or Marshall Island* or Mauritania* or Mauriti* or Mexic* or Micronesia* or Moldova* or Mongolia* or Montenegr* or Morocc* or Mozambi* or Myanma* or Burmese or Namibia* or Nepal* or Nicaragua* or Niger* or Nigeria* or Pakistan* or Palau* or Panama* or Papua New Guinea* or Paraguay* or Peru* or Philippines or Filipino or Romania* or Rwanda* or Samoa* or Sao Tome* or Senegal* or Serbia* or Seychell* or Sierra Leon* or Solomon Island* or Somalia* or South Africa* or Sudan* or Sri Lanka* or St Lucia* or St Vincent or Grenadines or Surinam* or Swazi* or Syria* or Tajikistan* or Tanzania* or Thai* or Timor* or Togo* or Tonga* or Tunisia* or Turk* or Turkmenistan* or Tuvalu* or Uganda* or Ukrain* or Uzbekistan* or Vanuatu* or Venezuela* or Vietnam* or West Bank or Gaza or Yemen* or Zambia* or Zimbabwe*).mp. |
| 15 | exp africa/ or exp caribbean region/ or exp central america/ or latin america/ or exp south america/ or asia/ or exp asia, central/ or exp asia, southeastern/ or exp asia, western/ or exp indian ocean islands/ or pacific islands/ or exp melanesia/ or exp micronesia/ or exp west indies/ |
| 16 | (africa* or asia* or caribbean or central america* or latin america* or south america* or melanesia* or micronesia* or polynesia*).tw. |
| 17 | (resource-limit* or resource-poor or low-resource* or limited-resource* or resource-constrain* or constrain*-resource* or under-resource* or poor*-resource* or resource-scarce* or scarce*-resource* or low-income or middle-income or lowincome or middleincome or LMIC*).tw. |
| 18 | ((developing or underdeveloped or under-developed or emerging or less-developed or least-developed or less-economically developed or least-economically developed or less-affluent or least-affluent) adj (country or countries or nation or nations or region or regions or economy or economies)).tw. |
| 19 | ((developing or underdeveloped or under-developed or less-developed or least-developed) adj world).tw. |
| 20 | (third-world* or thirdworld* or 3rd-world*).tw. |
| 21 | 13 or 14 or 15 or 16 or 17 or 18 or 19 or 20 |
| 22 | 12 and 21 |

**EMBASE**

| [# ▲](http://ovidsp.tx.ovid.com.ezproxy.lib.monash.edu.au/sp-3.31.1b/ovidweb.cgi?&S=KLIPFPKNAHDDLEJGNCEKDBLBPMJNAA00&Sort+Sets=descending) | **Searches** |
| --- | --- |
| 1 | (cost* or economic*).mp. |
| 2 | Diabetes Mellitus, Type 2/ or Hyperglycemia/ |
| 3 | hyperglyc*.mp. |
| 4 | hyperglycemia/ |
| 5 | (niddm or t2dm).mp. |
| 6 | (insulin resistance or insulin rasitant).mp. |
| 7 | T2D.mp. |
| 8 | ((adult-onset or ketosis-resistant or maturity-onset or non-insulin-dependent or noninsulin-dependent or slow-onset or type 2 or type ii) adj1 diabet*).mp. |
| 9 | (glucose or blood sugar or glyco* or glyca* or glyce*).mp. |
| 10 | ("hb a1*" or "hba1*" or hemoglobina1* or hemoglobin a1* or haemoglobina1* or haemoglobin a1*).mp. |
| 11 | 2 or 3 or 4 or 5 or 6 or 7 or 8 |
| 12 | 9 or 10 |
| 13 | 1 and 11 and 12 |
| 14 | Developing Countries/ |
| 15 | (Afghanistan* or Albania* or Algeria* or Angola* or Argentina* or Armenia* or Azerbaijan* or Bangladesh* or Belarus* or Beliz* or Benin* or Bhutan* or Bolivia* or Bosnia* or Herzegovin* or Botswan* or Brazil* or Bulgaria* or Burkina* or Burundi* or Cabo Verde* or Cape Verde* or Cambodia* or Cameroon* or Central African or Chad* or China or Chinese or Colombia* or Comor* or Congo* or Costa Rica* or Cote d'Ivoir* or Ivory Coast or Cuba* or Djibouti* or Dominica* or Ecuador* or Egypt* or El Salvador* or Eritrea* or Ethiopia* or Fiji* or Gabon* or Gambia* or Georgia* or Ghana* or Grenad* or Guatemala* or Guinea* or Guyan* or Haiti* or Hondura* or Hungar* or India* or Indonesia* or Iran* or Iraq* or Jamaica* or Jordan* or Kazakhstan* or Kenya* or Kiribati* or Korea* or Kosov* or Kyrgyz Republic or Lao* or Leban* or Lesotho* or Liberia* or Libya* or Macedonia* or Madagascar* or Malawi* or Malaysia* or Maldiv* or Mali* or Marshall Island* or Mauritania* or Mauriti* or Mexic* or Micronesia* or Moldova* or Mongolia* or Montenegr* or Morocc* or Mozambi* or Myanma* or Burmese or Namibia* or Nepal* or Nicaragua* or Niger* or Nigeria* or Pakistan* or Palau* or Panama* or Papua New Guinea* or Paraguay* or Peru* or Philippines or Filipino or Romania* or Rwanda* or Samoa* or Sao Tome* or Senegal* or Serbia* or Seychell* or Sierra Leon* or Solomon Island* or Somalia* or South Africa* or Sudan* or Sri Lanka* or St Lucia* or St Vincent or Grenadines or Surinam* or Swazi* or Syria* or Tajikistan* or Tanzania* or Thai* or Timor* or Togo* or Tonga* or Tunisia* or Turk* or Turkmenistan* or Tuvalu* or Uganda* or Ukrain* or Uzbekistan* or Vanuatu* or Venezuela* or Vietnam* or West Bank or Gaza or Yemen* or Zambia* or Zimbabwe*).mp. |
| 16 | exp africa/ or exp caribbean region/ or exp central america/ or latin america/ or exp south america/ or asia/ or exp asia, central/ or exp asia, southeastern/ or exp asia, western/ or exp indian ocean islands/ or pacific islands/ or exp melanesia/ or exp micronesia/ or exp west indies/ |
| 17 | (africa* or asia* or caribbean or central america* or latin america* or south america* or melanesia* or micronesia* or polynesia*).tw. |
| 18 | (resource-limit* or resource-poor or low-resource* or limited-resource* or resource-constrain* or constrain*-resource* or under-resource* or poor*-resource* or resource-scarce* or scarce*-resource* or low-income or middle-income or lowincome or middleincome or LMIC*).tw. |
| 19 | ((developing or underdeveloped or under-developed or emerging or less-developed or least-developed or less-economically developed or least-economically developed or less-affluent or least-affluent) adj (country or countries or nation or nations or region or regions or economy or economies)).tw. |
| 20 | ((developing or underdeveloped or under-developed or less-developed or least-developed) adj world).tw. |
| 21 | (third-world* or thirdworld* or 3rd-world*).tw. |
| 22 | 14 or 15 or 16 or 17 or 18 or 19 or 20 or 21 |
| 23 | 13 and 22 |

**CINAHL**

| S23 | S12 AND S21 |
| --- | --- |
| S22 | S12 AND S21 |
| S21 | S13 OR S14 OR S15 OR S16 OR S17 OR S18 OR S20 |
| S20 | AB third-world* or thirdworld* or 3rd-world* |
| S19 | AB developing world or underdeveloped world or under-developed world or less-developed world or least-developed world |
| S18 | AB ( developing and (count* or nation* or region* or econom*) ) OR AB ( underdeveloped and (count* or nation* or region* or econom*) ) OR AB ( under-developed and (count* or nation* or region* or econom*) ) OR AB ( emerging and (count* or nation* or region* or econom*) ) OR AB ( less-developed and (count* or nation* or region* or econom*) ) OR AB ( least-developed developing and (count* or nation* or region* or econom*) ) OR AB ( less-economically developed developing and (count* or nation* or r [...](javascript:showHistoryTerm('ctl00_ctl00_FindField_FindField_historyControl_HistoryRepeater_ctl05_ellipsis',true)) |
| S17 | AB resource-limit* or resource-poor or low-resource* or limited-resource* or resource-constrain* or constrain*-resource* or under-resource* or poor*-resource* or resource-scarce* or scarce*-resource* or low-income or middle-income or lowincome or middleincome or LMIC* |
| S16 | AB africa* or asia* or caribbean or central america* or latin america* or south america* or melanesia* or micronesia* or polynesia* |
| S15 | MW exp africa/ or exp caribbean region/ or exp central america/ or latin america/ or exp south america/ or asia/ or exp asia, central/ or exp asia, southeastern/ or exp asia, western/ or exp indian ocean islands/ or pacific islands/ or exp melanesia/ or exp micronesia/ or exp west indies |
| S14 | AB Afghanistan* or Albania* or Algeria* or Angola* or Argentina* or Armenia* or Azerbaijan* or Bangladesh* or Belarus* or Beliz* or Benin* or Bhutan* or Bolivia* or Bosnia* or Herzegovin* or Botswan* or Brazil* or Bulgaria* or Burkina* or Burundi* or Cabo Verde* or Cape Verde* or Cambodia* or Cameroon* or Central African or Chad* or China or Chinese or Colombia* or Comor* or Congo* or Costa Rica* or Cote d'Ivoir* or Ivory Coast or Cuba* or Djibouti* or Dominica* or Ecuador* or Egypt* or El Salva [...](javascript:showHistoryTerm('ctl00_ctl00_FindField_FindField_historyControl_HistoryRepeater_ctl09_ellipsis',true)) |
| S13 | MH Developing Countries |
| S12 | S1 AND S10 AND S11 |
| S11 | S8 OR S9 |
| S10 | S2 OR S3 OR S4 OR S5 OR S6 OR S7 |
| S9 | TX hb a1* or hba1* or hemoglobina1* or hemoglobin a1* or haemoglobina1* or haemoglobin a1* |
| S8 | TX glucose or blood sugar or glyco* or glyca* or glyce* |
| S7 | TX adult-onset diabet* or ketosis-resistant diabet* or maturity-onset diabet* or non-insulin-dependent diabet* or noninsulin-dependent diabet* or slow-onset diabet* or type 2 diabet* or type ii diabet* |
| S6 | TX T2D |
| S5 | TX insulin resistance or insulin resistant |
| S4 | TX niddm or t2dm |
| S3 | TX hyperglyc* |
| S2 | MH Diabetes Mellitus, Type 2 or Hyperglycemia |
| S1 | TX cost* or economic* |

**PSYCINFO**

| [# ▲](http://ovidsp.tx.ovid.com.ezproxy.lib.monash.edu.au/sp-3.31.1b/ovidweb.cgi?&S=KLIPFPKNAHDDLEJGNCEKDBLBPMJNAA00&Sort+Sets=descending) | **Searches** |
| --- | --- |
| 1 | (cost* or economic*).mp. |
| 2 | Diabetes Mellitus, Type 2/ or Hyperglycemia/ |
| 3 | hyperglyc*.mp. |
| 4 | (niddm or t2dm).mp. |
| 5 | (insulin resistance or insulin resistant).mp. |
| 6 | T2D.mp. |
| 7 | ((adult-onset or ketosis-resistant or maturity-onset or non-insulin-dependent or noninsulin-dependent or slow-onset or type 2 or type ii) adj1 diabet*).mp. |
| 8 | (glucose or blood sugar or glyco* or glyca* or glyce*).mp. |
| 9 | ("hb a1*" or "hba1*" or hemoglobina1* or hemoglobin a1* or haemoglobina1* or haemoglobin a1*).mp. |
| 10 | 2 or 3 or 4 or 5 or 6 or 7 |
| 11 | 8 or 9 |
| 12 | 1 and 10 and 11 |
| 13 | Developing Countries/ |
| 14 | (Afghanistan* or Albania* or Algeria* or Angola* or Argentina* or Armenia* or Azerbaijan* or Bangladesh* or Belarus* or Beliz* or Benin* or Bhutan* or Bolivia* or Bosnia* or Herzegovin* or Botswan* or Brazil* or Bulgaria* or Burkina* or Burundi* or Cabo Verde* or Cape Verde* or Cambodia* or Cameroon* or Central African or Chad* or China or Chinese or Colombia* or Comor* or Congo* or Costa Rica* or Cote d'Ivoir* or Ivory Coast or Cuba* or Djibouti* or Dominica* or Ecuador* or Egypt* or El Salvador* or Eritrea* or Ethiopia* or Fiji* or Gabon* or Gambia* or Georgia* or Ghana* or Grenad* or Guatemala* or Guinea* or Guyan* or Haiti* or Hondura* or Hungar* or India* or Indonesia* or Iran* or Iraq* or Jamaica* or Jordan* or Kazakhstan* or Kenya* or Kiribati* or Korea* or Kosov* or Kyrgyz Republic or Lao* or Leban* or Lesotho* or Liberia* or Libya* or Macedonia* or Madagascar* or Malawi* or Malaysia* or Maldiv* or Mali* or Marshall Island* or Mauritania* or Mauriti* or Mexic* or Micronesia* or Moldova* or Mongolia* or Montenegr* or Morocc* or Mozambi* or Myanma* or Burmese or Namibia* or Nepal* or Nicaragua* or Niger* or Nigeria* or Pakistan* or Palau* or Panama* or Papua New Guinea* or Paraguay* or Peru* or Philippines or Filipino or Romania* or Rwanda* or Samoa* or Sao Tome* or Senegal* or Serbia* or Seychell* or Sierra Leon* or Solomon Island* or Somalia* or South Africa* or Sudan* or Sri Lanka* or St Lucia* or St Vincent or Grenadines or Surinam* or Swazi* or Syria* or Tajikistan* or Tanzania* or Thai* or Timor* or Togo* or Tonga* or Tunisia* or Turk* or Turkmenistan* or Tuvalu* or Uganda* or Ukrain* or Uzbekistan* or Vanuatu* or Venezuela* or Vietnam* or West Bank or Gaza or Yemen* or Zambia* or Zimbabwe*).mp. |
| 15 | (africa* or asia* or caribbean or central america* or latin america* or south america* or melanesia* or micronesia* or polynesia*).tw. |
| 16 | (resource-limit* or resource-poor or low-resource* or limited-resource* or resource-constrain* or constrain*-resource* or under-resource* or poor*-resource* or resource-scarce* or scarce*-resource* or low-income or middle-income or lowincome or middleincome or LMIC*).tw. |
| 17 | ((developing or underdeveloped or under-developed or emerging or less-developed or least-developed or less-economically developed or least-economically developed or less-affluent or least-affluent) adj (country or countries or nation or nations or region or regions or economy or economies)).tw. |
| 18 | ((developing or underdeveloped or under-developed or less-developed or least-developed) adj world).tw. |
| 19 | (third-world* or thirdworld* or 3rd-world*).tw. |
| 20 | 13 or 14 or 15 or 16 or 17 or 18 or 19 or "20".mp. [mp=title, abstract, heading word, table of contents, key concepts, original title, tests & measures] |
| 21 | 12 and "21".mp. [mp=title, abstract, heading word, table of contents, key concepts, original title, tests & measures] |

**Appendix 2** Quality assessment tool

The consensus results of the quality assessment by two independent authors

| **Criteria** | |  |
| --- | --- | --- |
|  | **General** |  |
| 1 | Is the definition clear and precise? | 1 = the definition of the type of diabetes considered is clear and all the morbidities and co-morbidities considered are listed. |
|  |  | 0 = the definition is vague and do not include any details of all the morbidities and co-morbidities considered |
| 2 | Which complications the authors have included? | 1 = more than 4 complications are considered and specified. |
|  |  | 0,5 = up to 3 complications are considered for each patient but they are not specified. |
|  |  | 0 = no complications are considered or if they are considered there is no clear documentation in their inclusion. |
| **Sample** | |  |
| 3 | Are sources for population data reliable? | 1 = self-assessment and questionnaire are confirmed by hospital records or hospitals and practitioners bills. |
|  |  | 0,5 = The only sources of data are questionnaire and self-assessment. |
|  |  | 0 = The sources of data are not defined or are subject to a number of biases. |
| 4 | The period of study is appropriate? | A period of evaluation is considered appropriate if is equal or more than |
|  |  | 6 months for prevalence- based studies and consider more than 1 year for incidence based studies. |
| **Costs** | |  |
| 5 | Does the study include the relevant costs? | 1 = the costs included are relevant for the objective of the stud. (minimum of 80% of the costs included in the section costs of this table) |
|  |  | 0,5 = the inclusion of the costs is partial |
|  |  | 0 = there are missing a large number of costs that should be included or there is no specification of the costs included |
| 6 | Are the inclusion of the costs appropriate for the objective of the study? | 1 = considering the aim, all the necessary type of costs are included. (for ex for the evaluation of direct costs of a drug treatment all the costs borne by the patients directly and by the health care are included) |
|  |  | 0,5 = Only partial relevant costs are included. There are missing of some important costs related to the aim of the study. |
|  |  | 0 = Although the study aim is to consider a general costs of diabetes or a costs of drug or complications there are included only a category of costs (for ex direct costs). |
| 7 | Has the Diabetes severity Index been used? | 1 = Yes |
|  |  | 0 =No |
| 8 | Is adequate documentation and justification given for cost components, data and sources, assumptions and methods? | 1 = detailed justifications are given for all the approach and methods adopted. The exclusion and inclusion of categories of cost and data are well motivated. All the sources are documented |
|  |  | 0,5 = partial justification is given for the methods and approach adopted. There is limited or absence of justifications for the inclusion or exclusion of costs. The documentation is scarce and not precise. |
|  |  | 0 = absence or minimal presence of documentation and justification |
| 9 | Are important limitations discussed regarding the cost components, data, assumptions and methods? | 1 = all the most important limitations are discussed. In same cases some minor limitation is discussed. |
|  |  | 0,5 = one or only not important limitations are discussed. |
|  |  | 0 = there is no discussion around the limitations of the study. |
| **Methods** | |  |
| 10 | Is the data representative of the study population? | 1 = prevalence-based |
|  |  | 0, 5 = Incidence based |
|  |  | 0 = no definition of the approach considered |
| 11 | Was the approach appropriate? | 1 = bottom-up approach. |
|  |  | 0,5 = top down. |
|  |  | 0 = no approach defined/ or impossibility to infer the approach employed |
| 12 | Is the estimation method of the cost of diabetes appropriate? | 1 = Incremental costs method. |
|  |  | 0,5 = Total disease costs |
|  |  | 0 = no methods designed or impossibility to retrieved a clear method from the study. |
| 13 | Are the deviation standard and the means calculated? | 1 = both, standard deviation and Means are calculated. 0,5: only one of them is calculated. |
|  |  | 0: none of them is calculated |
| 14 | Is a sensitivity analysis performed? | 1 = the sensitivity analysis is performed and the results are clearly shown. |
|  |  | 0,5 = some linear regression method are employed to correlate the variables |
|  |  | 0 = no sensitivity analysis or linear regression are performed. |
| 15 | Which statistical methods are used | 1 = the statistical analysis is performed with consistent statistical formulas. The formulas used should non-parametrical statistical hypothesis test. |
